# Intraligamentary anaesthesia: a local anaesthesia technique in equine dentistry

**DOI:** 10.1186/s13028-025-00836-3

**Published:** 2025-11-26

**Authors:** Stijn Teysen, Wouter Demey, Robert Menzies, Carsten Staszyk, Dowen Birkhed, Torbjörn Lundström

**Affiliations:** 1 DAP Vetrident, Geertskouter 32, Asse, 1730 Belgium; 2 DAP Equide, Stappersestraat 3, Schaffen, 3290 Belgium; 3 Complete Veterinary Dental Care Pty Ltd, 132 Naroghid Road, Camperdown, VIC 3260 Australia; 4 Faculty of Veterinary Medicine, Justus-Liebig-University Giessen, Frankfurter Str. 98, 35392 Gießen, Germany; 5 Fersens Väg 14B, SE-211 42 Malmö, Sweden; 6 Djurtandvårdskliniken, Västra Husby, Norrköping, 605 96 Sweden

**Keywords:** Extraction, Nerve block, Periodontal ligament injection

## Abstract

**Background:**

The injection of a local anaesthetic into the periodontal ligament (PDL) to achieve desensitisation of the pulp, periodontium, and adjacent tissues is a well-established technique in human dentistry, particularly in paediatric patients. This approach, commonly referred to as intraligamental or intraligamentary anaesthesia (ILA), has been widely adopted due to its effectiveness and relative simplicity. The aims of the present publication were: (1) to provide a review of the literature, (2) to describe an ILA technique adapted by the authors for use in equine dentistry, and (3) to evaluate this technique in an equine cadaver study.

**Results:**

Following injection of the solution into the periodontal ligament, the solution was observed to spread extensively through the PDL and alveolar bone, accumulating around the apex of the tooth. In horses, this distribution appeared to occur via both diffusion along the PDL within the periodontal space and infiltration into bone marrow spaces.

**Conclusion:**

The described ILA technique is straightforward to perform and can practically be applied in equine dental procedures. The cadaver study demonstrated consistent distribution of the injected solution around the root apex, supporting the anatomical basis for potential desensitisation of the pulp, periodontium, and surrounding tissues. While clinical studies are needed to confirm efficacy, these findings indicate that ILA may represent a useful additional technique for achieving local anaesthesia prior to tooth extraction in horses.

## Background

The injection of local anaesthetics into the periodontal ligament to achieve desensitisation of the pulp, periodontium and adjacent tissues, is a well-established technique in humans, especially in paediatric dentistry [1], but also in adult patients [2]. The technique is commonly referred to as “intraligamental/intraligamentary anaesthesia” (ILA), or periodontal ligament injection.

In equine dentistry, the technique has not been described in detail. It has however been used by one of the authors (TL) in his clinic for equine dentistry for over 30 years. The purpose of the present article was to provide a review of the literature regarding ILA and to describe the technique proposed by the authors in an equine cadaver study.

Equine dentistry has advanced in recent decades as a result of a better understanding of the anatomophysiological aspects of the equine oral cavity, the availability of dedicated dental instruments, the inclusion of routine oral examinations and the development and improvement of a variety of oral surgical techniques. In addition, there is a growing interest in minimally invasive procedures in equine dentistry, which are carried out on the standing, sedated horse [3], so that risky general anaesthesia can be avoided.

In the field of equine dentistry and in oral and facial surgery, standing sedation is routinely combined with perineural nerve blocks. Techniques for blocks of the infraorbital nerve within the depth of pterygopalatine fossa and of the alveolar inferior nerve at the mandibular foramen underneath the medial pterygoid muscle have been extensively described in previous articles [4, 5]. Although the perineural nerve blocks were described as relatively simple and safe, some disadvantages and risks remain due to the equine specific anatomy. Reported complications after block of the infraorbital include: hematoma formation, retrobulbar abscess formation, ocular protrusion, corneal ulceration, blindness, neuropraxia, cellulitis and meningitis [4–7]. After block of the alveolar inferior nerve self-inflicted lingual trauma and oral abscess formation were reported [8]. The most relevant disadvantage is certainly the persistence of partial pain sensation, as the methods described cannot completely block all pain-conducting nerve fibers.

Therefore, for painful interventions, local supplementary anaesthesia of the region of interest is often desired and is even more frequently a prerequisite. Local anaesthesia is used to desensitize the surgical area, alleviate perioperative pain, and decrease the amount of sedation needed to work safely and efficiently [2]. The development of safer and more efficient local anaesthetic agents, the use of vasoconstrictors, and improvements in administration techniques have further enhanced the efficacy and safety of local anaesthesia [9, 10].

In human dentistry, the risk of complications and the relatively high number of failures, especially of the inferior alveolar nerve block (IANB), encouraged dentists to further develop alternative anaesthetic techniques for orofacial procedures [11]. ILA was introduced in human dentistry in the early 20th century [12]. It gained popularity in the 1970s due to the development of a high-pressure dental syringe which accommodates a cartridge that contains 1.8 mL of anaesthetic solution: Ligmaject and Peripress Pen [13, 14].

Intraligamentary injections in human dentistry are usually made with a 30-gauge short disposable needle. The needle is inserted via the gingival sulcus into the PDL. The bevel of the needle faces the alveolar wall. Classically four injection sites are chosen: mesiobuccal, mesiolingual (or -palatal), distobuccal and distolingual (or -palatal). The needle is inserted until resistance is met (2–3 mm) [15]. The total amount of anaesthetic varies between 0.2 and 1.8 mL depending on the number of injections, the specific tooth and the type of procedure. The anaesthetic solution is delivered slowly with a manual dental pressure syringe or via a computerised syringe. The use of computer-assisted devices to administer a local anaesthetic at a set speed and pressure further decreases the discomfort during the injection [16]. The devices inject local anaesthesia over a period of 1–3 min.

As in human dentistry, veterinarians further develop anaesthetic techniques to reduce the complications and improve the accuracy of the local anaesthetics. Ultrasound-guided local techniques are reported and can be used [17]. The ILA-technique might be a valuable alternative to nerve blocks in the equine patient. In human dentistry, ILA is a widely used, efficient and clinically-safe anaesthetic technique [18–20]. To the authors’ knowledge, the ILA-technique has not been thoroughly described in horses, probably due to the more difficult approach and the enormous length of the equine periodontal space.

## Methods

### Equipment and technique

**Fig. 1:**
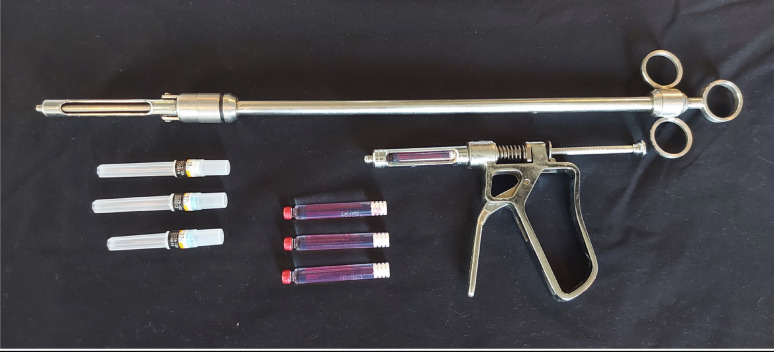
Instrumentation for ILA in the horse. A 27G x 35 mm dental needle is used in combination with a long-handled syringe. For ILA of incisors, wolf teeth, canine teeth or second premolars a dental pressure syringe can be used. Both syringes accommodate a 1.8mL cartridge with local anaesthetic

In the molar region, a long-handled syringe [40 cm Extended Intra-Oral LA Syringe, Equine Blades Direct Ltd, Wedmore, United Kingdom] in combination with a 27G × 35 mm dental needle is used. The long-handled syringe accommodates a cartridge that contains 1.8 mL of anaesthetic solution. In the more rostral part of the oral cavity, a high-pressure dental syringe, which accommodates the same cartridges can be applied in combination with the same dental needles (Fig. 1).

**Fig. 2:**
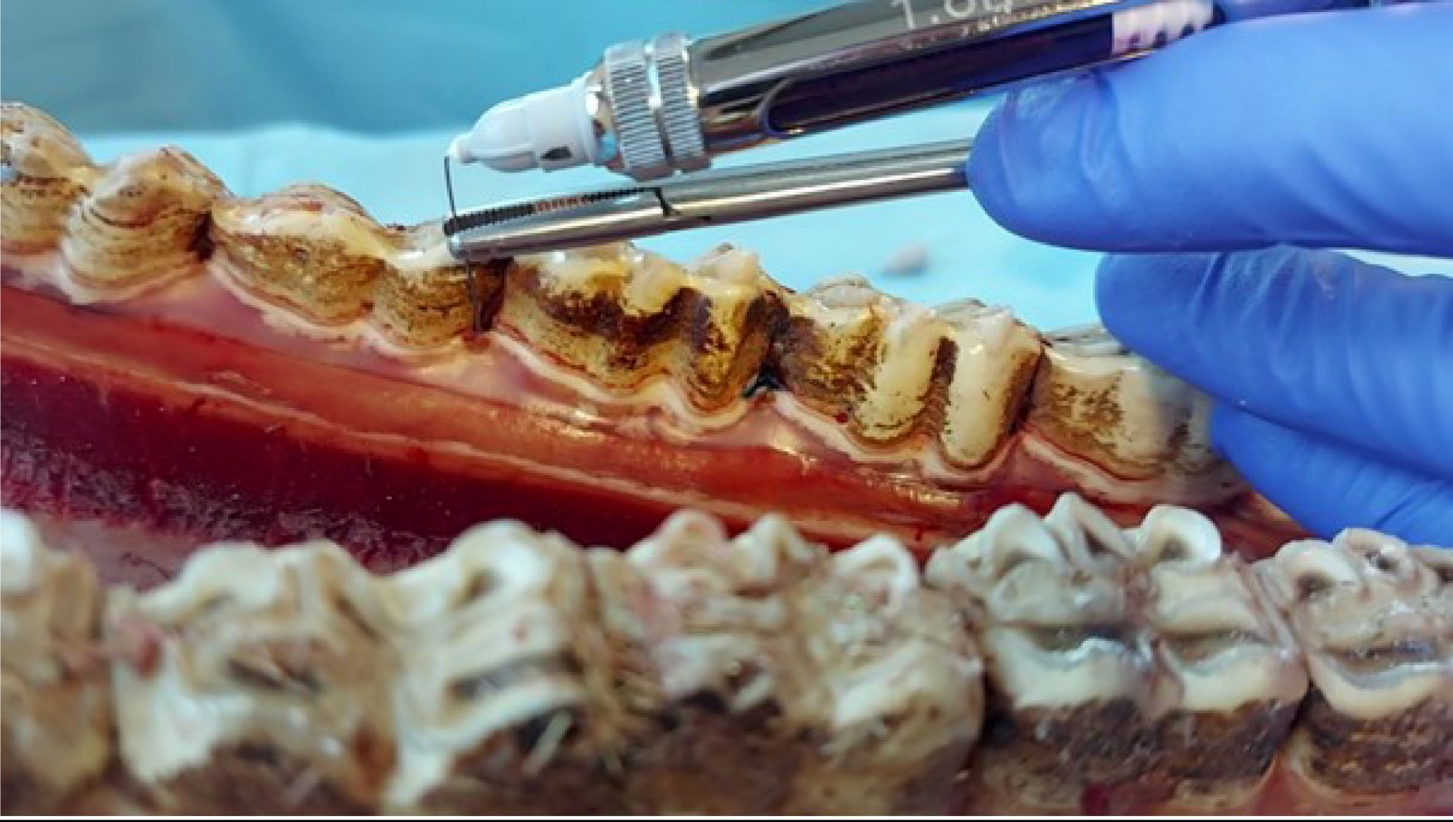
Positioning of the needle for performing an intraligamentary injection. The needle is directed into the periodontal ligament with the help of a needle holder or a crocodile forceps. The needle is directed towards the tooth of interest

**Fig. 3:**
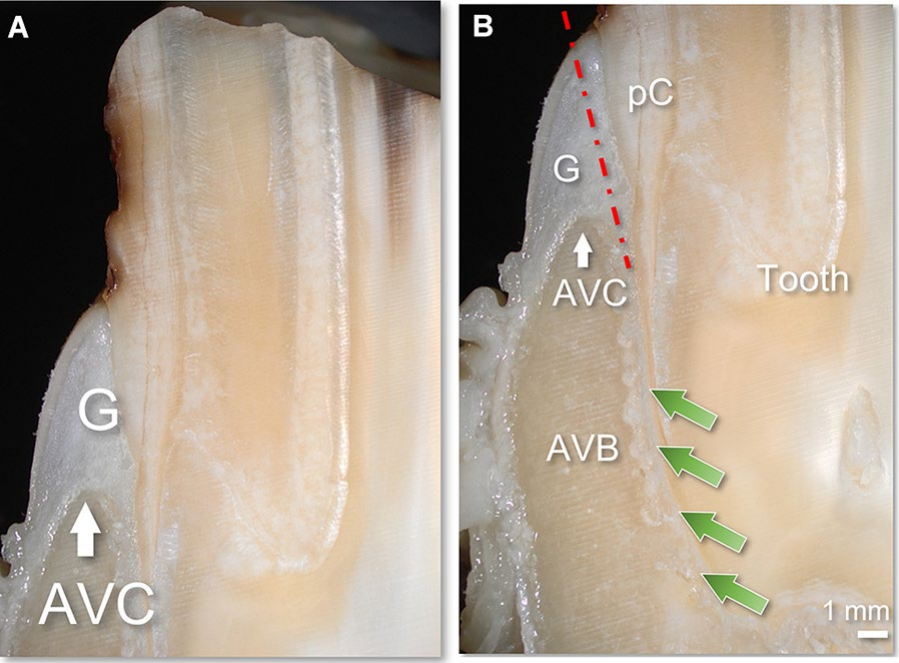
Stereomicroscopic images of a longitudinal section of a lower cheek tooth and its surroundings. The gingiva (G) rises up for approximately 5 mm above the alveolar crest (AVC). The bulk of peripheral cementum (pC) requires a slight angulation of an injection needle (red dotted line) towards the tooth to advance the needle in the PDL (green arrows), in between the tooth and the alveolar bone (AVB)

**Fig. 4:**
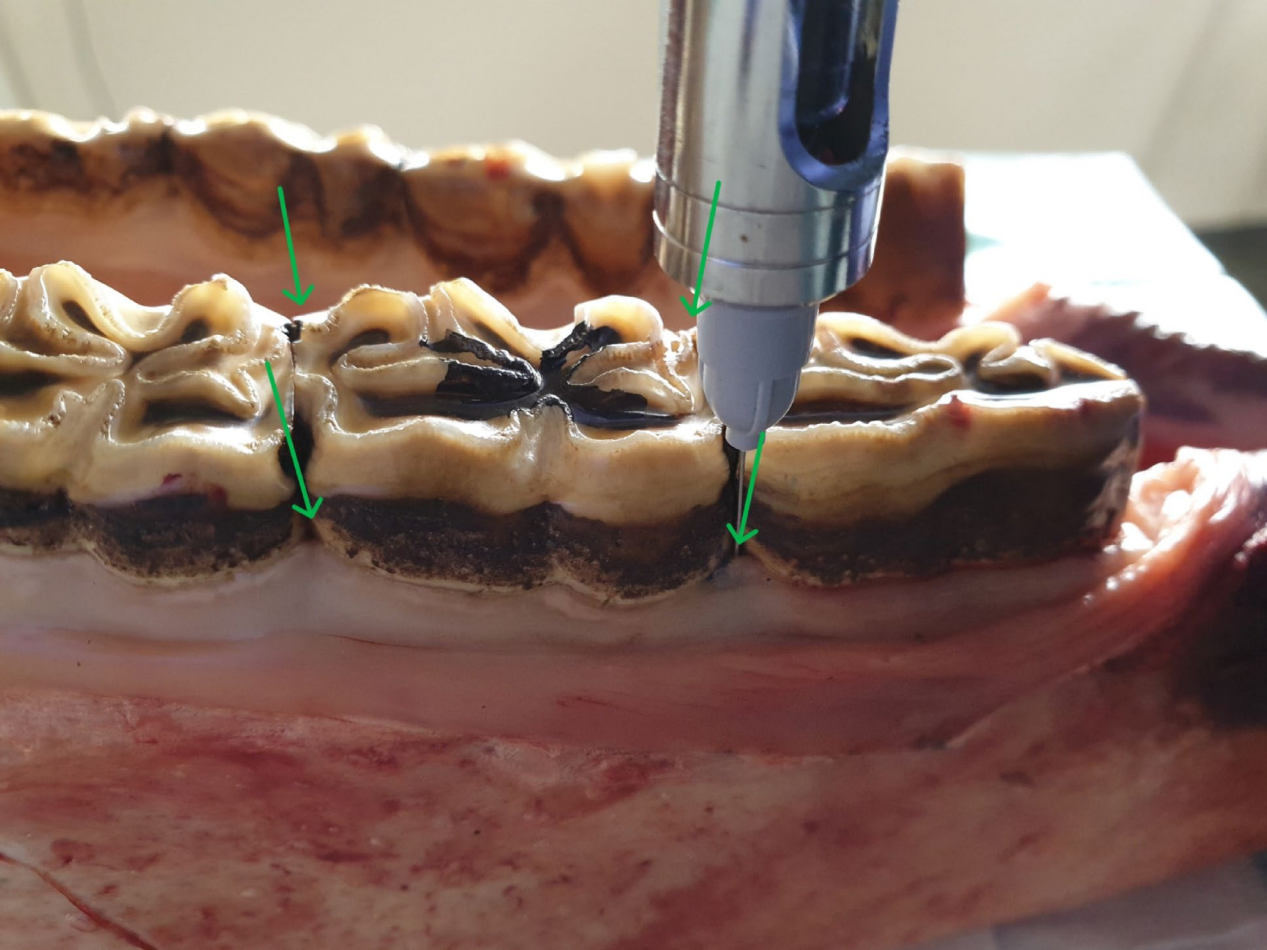
Injection sites for a lower cheek tooth. The needle is directed towards the tooth and is inserted into the periodontal ligament until resistance is met (in general after 25–35 mm). The procedure is repeated on the mesiobuccal, mesiolingual (or -palatal), distobuccal and distolingual (or -palatal) side (green arrows). In general, one cartridge of anaesthetic solution is administered at each of the four injection sites

With the help of crocodile forceps or a long needle holder, the needle is directed into the PDL (Fig. 2). The needle is inserted into the periodontal ligament until resistance is met (in general after 25–35 mm). Although the purpose is not to advance the needle to the apical area, it is often possible to introduce the entire 35-mm needle into the PDL within the periodontal space (Fig. 3). After injecting the solution, the procedure is repeated on the mesiobuccal, mesiolingual (or -palatal), distobuccal and distolingual (or -palatal) sides (Fig. 4). Where this is not possible, molar spreader forceps may be used very gently to create more space to allow correct needle placement. In the authors’ experience, this is only necessary on rare occasions, with a higher prevalence in older horses due to the more rigid organisation of the PDL.

### Cadaver study

Four equine cadaver heads that had been previously frozen and thawed immediately prior to the study, as well as two fresh equine heads, were used to assess the distribution of local anaesthetic following intraligamentary injection. On each skull, four cheek teeth were randomly selected. Injections were performed at the four designated sites as described above. Half of the injections were performed with a high-pressure dental syringe; the other half were done with a long-shafted dental syringe. At each site, one cartridge (1.8 mL) of black ink was manually administered. Care was taken not to inject with too high pressure, especially when using the pressure syringe. Each of the injections took 30 s or more.

Immediately after injection, the skulls were sectioned using a band saw without water cooling. Transverse sections were made to include the tooth of interest along with the adjacent teeth rostral and caudal to it. A minimum of three transverse sections were made for each treated tooth. The first section was made 1 cm below the gumline, the second section was made halfway along the estimated reserve crown, and the third section was made at the apical region. Photographs were taken of each section, and the distribution of black ink was evaluated macroscopically.

## Results

**Fig. 5:**
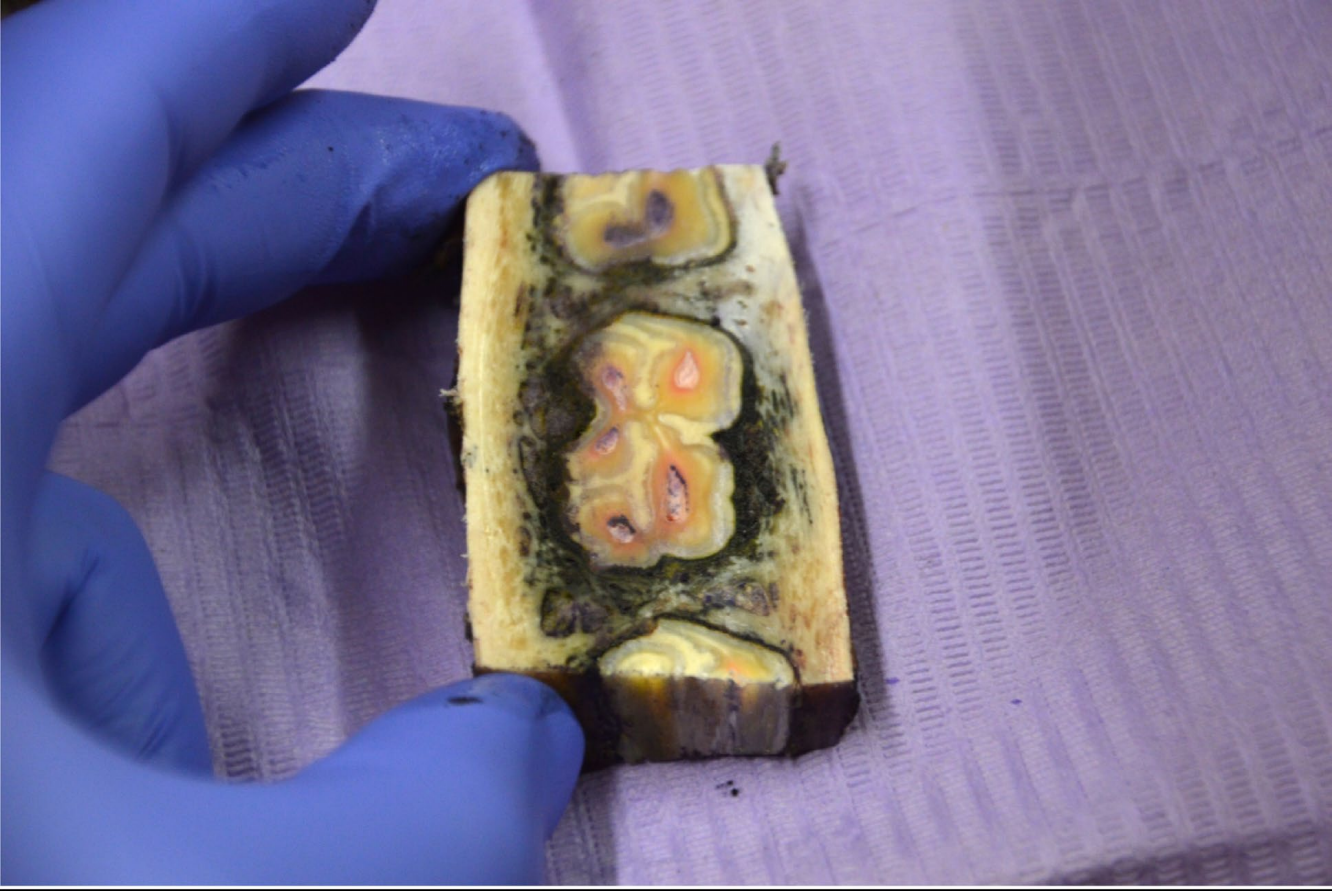
Equine lower cheek tooth after intraligamentary injections. Transverse section approximately 4 cm apically from the gingival margin, immediately after performing intraligamentary injections at the 4 described sites. Although the ink was injected into the PDL, the fluid did not stay within this structure. Ink was seen in the PDL as well as in the surrounding alveolar bone

Although the ink was injected into the PDL within the periodontal space, the fluid did not stay within this structure. Ink was seen in the PDL as well as in the surrounding alveolar bone (Fig. 5). It is most likely that the ample fenestrations in the alveolar bone wall are the route of diffusion for the solution from the PDL [21]. The injected solution was found pooling around the apex of the tooth, but spread much further than presumed. In all sections that were made, ink was found around the apices of neighbouring teeth.

**Fig. 6:**
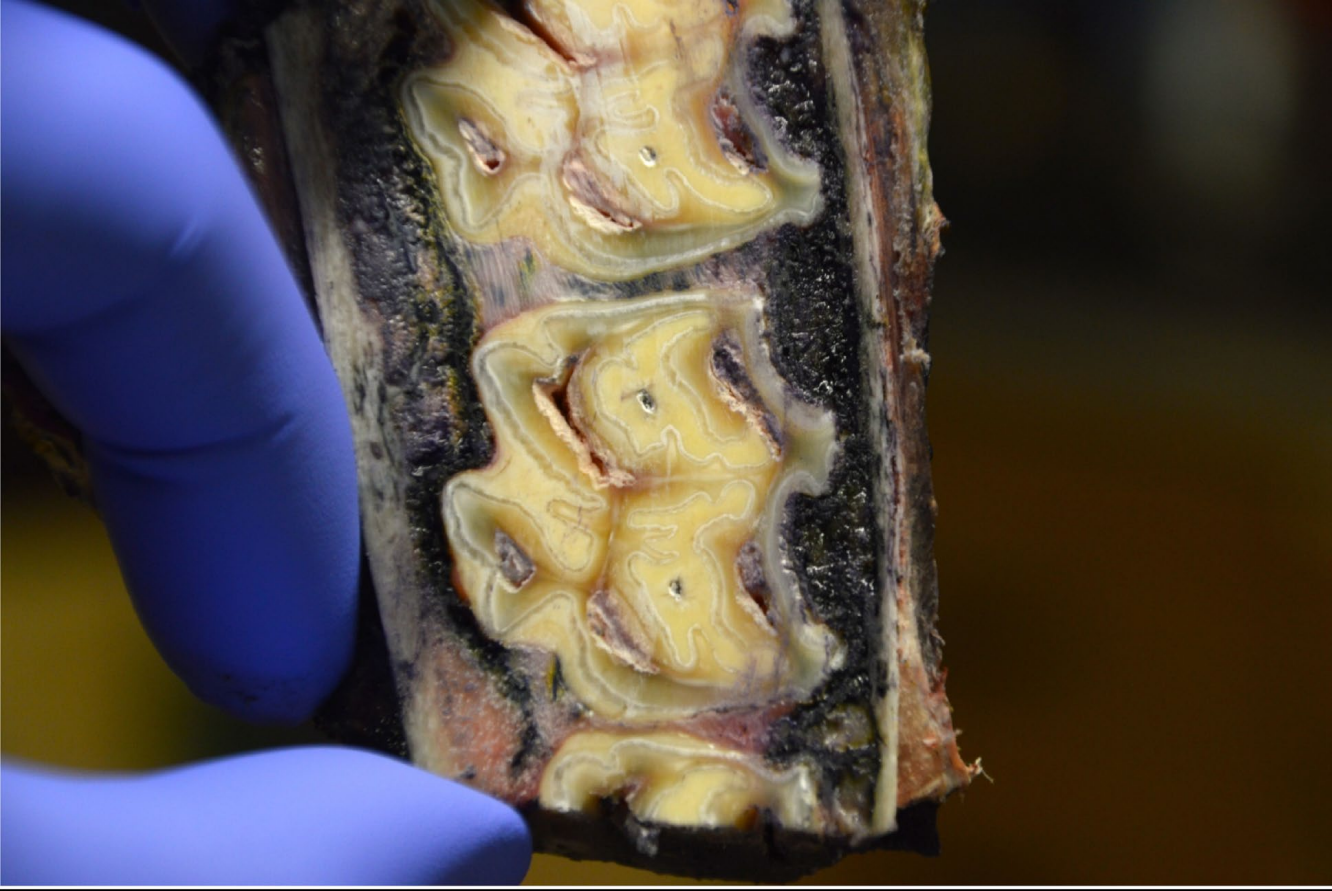
Equine upper cheek tooth after intraligamentary injections. Transverse section approximately 2 cm apically from the gingival margin, immediately after performing intraligamentary injections at the 4 described sites. A different pattern of diffusion was noticed between the upper and lower cheek teeth. Due to the less compact organization of the maxillary bone, a wider spread of ink was noticed compared to the lower cheek teeth. In both upper and lower cheek teeth ink was less present in the more occlusal part of the PDL, and the ink pooled around the apex of the tooth

A different pattern of diffusion was noticed between the upper and lower cheek teeth (Fig. 6). A wider spread of ink was noted around the upper cheek teeth, while ink remained in closer proximity to the tooth in the lower jaw.

No difference was observed between thawed and fresh specimens. Neither was there a difference in spread between the use of the extended syringe or the pressure gun.

## Discussion

The tooth and its surrounding tissues receive their innervation from the sensory branches from the fifth cranial nerve (trigeminal nerve) and the sympathetic fibres from the cervical ganglion [22]. All nerve fibres enter the tooth through the apical foramen. The sensory nerve bundles enter the PDL through several slits in the alveolar bone, located at the bottom and in the lateral wall of the alveolar socket [23].

Beside the dental and periodontal innervation provided by branches of the infraorbital and alveolar inferior nerves, which reach the tooth and its periodontal surrounding via a bony canal, additional nerve branches reach the periodontal space via the gingiva [24]. The nasopalatine nerve and the major palatine nerve provide branches for the palatal sides of the maxillary teeth, the lingual nerve provides branches for the lingual sides of the mandibular teeth. The mentioned nerves are most likely blocked by a perineural block of the maxillary nerve at the maxillary foramen or by a block of the mandibular nerve at the mandibular foramen, respectively. However, the buccal gingiva of the maxillary as well as of the mandibular cheek teeth is reached by branches of the buccal nerve, which is not desensitised by a perineural nerve block. Although no studies exist which evaluated the amount of periodontal sensitivity provided by the buccal nerve, it is assumed that reported remaining sensitivity after classical perineural blocks are provided by branches of the buccal nerve. Therefore, an intraligamentary injection of anaesthetics would at least supplement and complete a perineural block.

The injected local anaesthetic spreads through the PDL and alveolar bone and pools around the apex of the tooth. Data available from human dentistry [25–27] and the clinical experience of the authors confirm that the deposition of a volume of local anaesthetic around the apex of the tooth, injected into the PDL, will desensitize the tooth and its surrounding. In a human contrast studies the spread of the solution was noticed apically through the alveolar bony wall and bone marrow spaces, avoiding the PDL route [19]. Our observations concur with this route of distribution, although in most sections that were made, ink was also found in the PDL surrounding the teeth. Therefore, in horses, the spread of the injected solution is most likely a combination of the PDL route and the bone marrow route.

Since the reserve crown and roots are much longer in equine patients compared to human patients, the needle is advanced in the PDL as far as possible. In contrast to human dentistry, where this is only possible for a few millimetres, in horses it is often possible to advance the whole needle into the PDL, i.e. 35 mm. After the injection, ink was distributed in all aspects of the periodontal ligament and accumulated in the periapical area.

Due to the physical trauma of needle placement and the possible ischemia after injecting a local anaesthetic with a vasopressor, the ILA-technique should not be recommended for tooth-saving procedures without further research. In pigs and monkeys a local, mild and reversible inflammation can be observed within 24 h after injecting [28]. In most of the specimens all damage disappeared within eight days following injection. At 15 days following injection, all damage was considered healed, but in a very limited amount of samples some granulation tissue was still present in the lamina propria of the gingiva [28, 29]. An osteoclastic reaction and reversible bone resorption occur after injecting fluid under pressure in the PDL of dogs [30]. After 25 days the situation was normalised again. Slight postoperative localized pain due to periodontitis was reported by five human patients out of 187 after ILA. The pain disappeared after two days [15]. This has not yet been reported in equine patients; however, further clinical research is warranted.

The possible introduction of bacteria originating from the oral flora should be taken into consideration when performing an ILA. In children, 96.6% of the injections led to a transient bacteriemia. By comparison, toothbrushing alone caused a bacteraemia in 38.5% of the occasions [31]. In the equine patient a transient bacteraemia is encountered in 90% of the patients during a tooth extraction [32]. The same authors concluded that although arguments exist for prophylactic antimicrobial drugs administration prior to dental extractions, the use of antimicrobials should be restricted to cases in which it is deemed necessary [32]. The same principle applies for intraligamentary injections. Removing tartar, dental plaque and disinfection of the injection site with chlorhexidine might reduce the bacteraemia and inflammation post injection.

For reasons mentioned above, an increased risk of alveolar bone sequestration after tooth extraction might be a possible complication. However, in humans, there was no statistically significant difference in post-extraction dry socket formation between a perineural nerve block and an ILA [33]. The overall negative effect of the intraligamentary injection on the surrounding tissues seems to be mild and short lived [34]. In the primary dentition, ILA does not increase the risk of developmental disturbances to the underlying dental bud [35].

With increasing age, the collagen fibre bundles of the PDL become thicker, the collagen fibre arrangement becomes denser, and the portion of cemento-alveolar fascicles increases [36]. In older horses, the more rigid and dense organization of the PDL influences the needle placement when performing an ILA. On rare occasions, it might be necessary to use interdental molar spreaders to gently create space for the introduction of the needle into the PDL.

The most significant disadvantage of the ILA might be the shorter duration of action. In human dentistry, the technique is specifically used for procedures of less than 30 min. The presence of a vasoconstrictor seems to play an important role in the quality and duration of the anaesthesia as indicated by several studies [34, 37]. However, there is insufficient high-quality evidence to prove the superiority of one specific formulation over another as stated by a recent Cochrane review [38]. Increasing the administered volume does increase the success rate in humans [39]. The significantly larger volume used in horses might play a role in the prolonged effect that the authors have observed clinically.

In the cadaver study, a different pattern of diffusion was noticed between the upper and lower cheek teeth, due to the more compact organisation of the mandibular bone. The local anaesthetics will spread much further in the upper jaw than in the lower jaw, if the operator does not pay attention to the pressure while injecting.

In human dentistry ILA is referred to as a ‘single tooth anaesthesia’. The spreading of ink in the cadaver study does suggest that analgesia may be more widespread and not limited to a single tooth. However, the anaesthesia is more specific than a perineural nerve block. This has many advantages, including reducing the number of possible types of complications. Particularly for the anaesthesia of a mandibular molar, there is no risk of self-inflicted trauma to the tongue, due to desensitization of the lingual nerve.

The downside of the more specific anaesthesia of ILA is most obvious during the spreading phase of a tooth extraction. Despite a complete desensitization of the PDL of the diseased tooth, the horse might still react when placing molar spreaders in between the teeth. Pressure during spreading is probably felt through the complete arcade and not just on one tooth. Therefore, spreading needs to be done gently and with little pressure.

## Conclusions

In human dentistry, ILA is a widely used, efficient and clinically safe anaesthetic technique [2, 40]. Probably due to the length of the equine teeth and the more difficult approach, especially in the caudal region of the mouth, the ILA technique has never been thoroughly described in horses.

However, the described ILA technique is straightforward to perform and can be applied in equine dental procedures. The cadaver study demonstrated consistent distribution of the injected solution around the tooth apex, supporting the anatomical basis for potential desensitisation of the pulp, periodontium, and surrounding tissues after injecting a local anaesthetic.

Many of the possible complications that can occur with perineural nerve blocks can be avoided with ILA. Important limitations of the ILA technique are the more difficult access in older horses due to the more rigid organisation of the PDL, and the possibly shorter duration of action. Introducing the needle into the PDL and administering an anaesthetic solution might create a (transient) periodontal inflammation. Therefore, ILA is not recommended for tooth saving procedures without further research.

Based on the human literature and the findings in this cadaver study, ILA might be a possible technique to desensitise the pulp, periodontium, and surrounding tissues prior to tooth removal.

## Data Availability

The datasets used and/or analysed during the current study are available from the corresponding author on reasonable request.
